# Brain tumor image segmentation based on improved FPN

**DOI:** 10.1186/s12880-023-01131-1

**Published:** 2023-10-30

**Authors:** Haitao Sun, Shuai Yang, Lijuan Chen, Pingyan Liao, Xiangping Liu, Ying Liu, Ning Wang

**Affiliations:** 1 Department of Radiotherapy Room, Zhongshan Hospital of Traditional Chinese Medicine, ZhongShanGuangdong Province, 528400 China; 2 Department of Radiotherapy and Minimally Invasive Surgery, The Cancer Center of The Fifth Affiliated Hospital of Sun Yat-Sen University, Zhuhai, 519020 China; 3 Department of the Radiotherapy, The Fifth Affiliated Hospital of Guangzhou Medical University, Guangzhou, 510060 China

**Keywords:** Full convolutional neural network, U-Net model, Improved FPN model, Brain tumor segmentation

## Abstract

**Purpose:**

Automatic segmentation of brain tumors by deep learning algorithm is one of the research hotspots in the field of medical image segmentation. An improved FPN network for brain tumor segmentation is proposed to improve the segmentation effect of brain tumor.

**Materials and methods:**

Aiming at the problem that the traditional full convolutional neural network (FCN) has weak processing ability, which leads to the loss of details in tumor segmentation, this paper proposes a brain tumor image segmentation method based on the improved feature pyramid networks (FPN) convolutional neural network. In order to improve the segmentation effect of brain tumors, we improved the model, introduced the FPN structure into the U-Net structure, captured the context multi-scale information by using the different scale information in the U-Net model and the multi receptive field high-level features in the FPN convolutional neural network, and improved the adaptability of the model to different scale features.

**Results:**

Performance evaluation indicators show that the proposed improved FPN model has 99.1% accuracy, 92% DICE rating and 86% Jaccard index. The performance of the proposed method outperforms other segmentation models in each metric. In addition, the schematic diagram of the segmentation results shows that the segmentation results of our algorithm are closer to the ground truth, showing more brain tumour details, while the segmentation results of other algorithms are smoother.

**Conclusions:**

The experimental results show that this method can effectively segment brain tumor regions and has certain generalization, and the segmentation effect is better than other networks. It has positive significance for clinical diagnosis of brain tumors.

## Introduction

Glioma is the most common primary brain tumor. In adults, glioma accounts for 30% to 40% of all brain tumors and 80% of brain malignant tumors [1]. Surgical resection is the most effective treatment method at present, and then radiotherapy is used to treat some residual tumors and cells around the tumor focus, so that glioma can be well controlled [2]. The automatic segmentation of brain tumors is helpful to measure tumor characteristics and help doctors diagnose, plan treatment and predict survival in clinical applications [3].

Magnetic resonance imaging (MRI) is a commonly used technology in the field of radiology. Because of its advantages of non-invasive, non-ionizing radiation and high contrast of soft tissue imaging, it has become the preferred imaging method for brain tumor diagnosis and treatment [4]. At present, the gold standard of brain tumor segmentation is still manual segmentation, but it is expensive, time-consuming and subjective [5]. Therefore, a fast and accurate automatic segmentation method for brain tumor MRI is of great significance for clinical application.

At present, there are two main methods for automatic segmentation of brain tumor images. (1) Machine learning method based on artificial features. This method uses different classifiers for various artificial features, such as support vector machines with spatial and intensity features and Gaussian mixture models with intensity features. However, these algorithms require manual feature extraction, which is costly and error prone, and the model based on manual features is not generalized enough. (2) Based on end-to-end deep learning method. This method can achieve more accurate segmentation without designing complex manual features. For example, the full convolutional network (FCN) expands image level classification to pixel level classification, replaces the full connection layer with convolutional layer, and achieves better segmentation effect, becoming the pioneer of deep learning in semantic segmentation applications [6–9]. Its disadvantage is that it is insensitive to detail features, does not fully consider the relationship between pixels, and the segmentation result is not fine enough.

U-Net network model is an improvement and extension of FCN. It uses cascading operations to fuse depth features and shallow features, replacing the summation method in FCN. It alleviates the loss of deep features in the upsampling process, and effectively improves the segmentation accuracy [10]. V-Net is a segmentation application method for 3D medical images. The residual network is used in the coding part to ensure the deepening of the network depth and reduce the risk of gradient disappearance or gradient explosion. Daimary et al. proposed a hybrid depth neural network model, Seg UNet and U-SegNet, for brain tumor MRI image segmentation by combining SegNet and U-Net [11]. Zhu et al. discussed the application of the attention gate model in MRI brain tumor segmentation, and proposed a new model, AGResU-Net, to enhance feature learning and extract cerebellar tumor information [12]. Chen et al. proposed a new network VoxResNet, which seamlessly integrates multimodal and multi-level context information into the network to make use of complementary information of different modes and features of different scales [13]. Weng et al. [14] reviewed Generative Adversarial Network based fast MRI segmentation methods and shown that the model has good generalizability and robustness. U-Net proposed by Ronnebergeret al. [15] with the gated recurrent unit (GRU) on ahigher feature level to propagate slice features from one to another. The U-Net +  + network uses a series of grid like dense hop paths to compensate for the semantic difference between the encoder and decoder sub paths, so that the proportion difference of the feature map is smaller during fusion, the gradient flow is improved, and the segmentation accuracy is improved. However, the above-mentioned U-Net and U-Net +  + networks cannot extract the characteristic information of different receptive fields, and their multi-scale processing capacity is limited.

In view of the above shortcomings, this paper proposes a brain tumor image segmentation based on improved feature pyramid networks (FPN) convolution neural network, which can be applied to tumors of different sizes to achieve fine segmentation of brain tumor edge regions. The main contribution of this paper is to introduce the feature pyramid FPN in the downsampling phase of the U-Net network to obtain multi-scale context information with multiple receptive fields and improve the multi-scale processing capability of the network; It enriches the information required by each pixel in the classification, and alleviates the problem of poor edge segmentation of the above model target [16].

## Materials and methods

### U-Net model

**Fig. 1:**
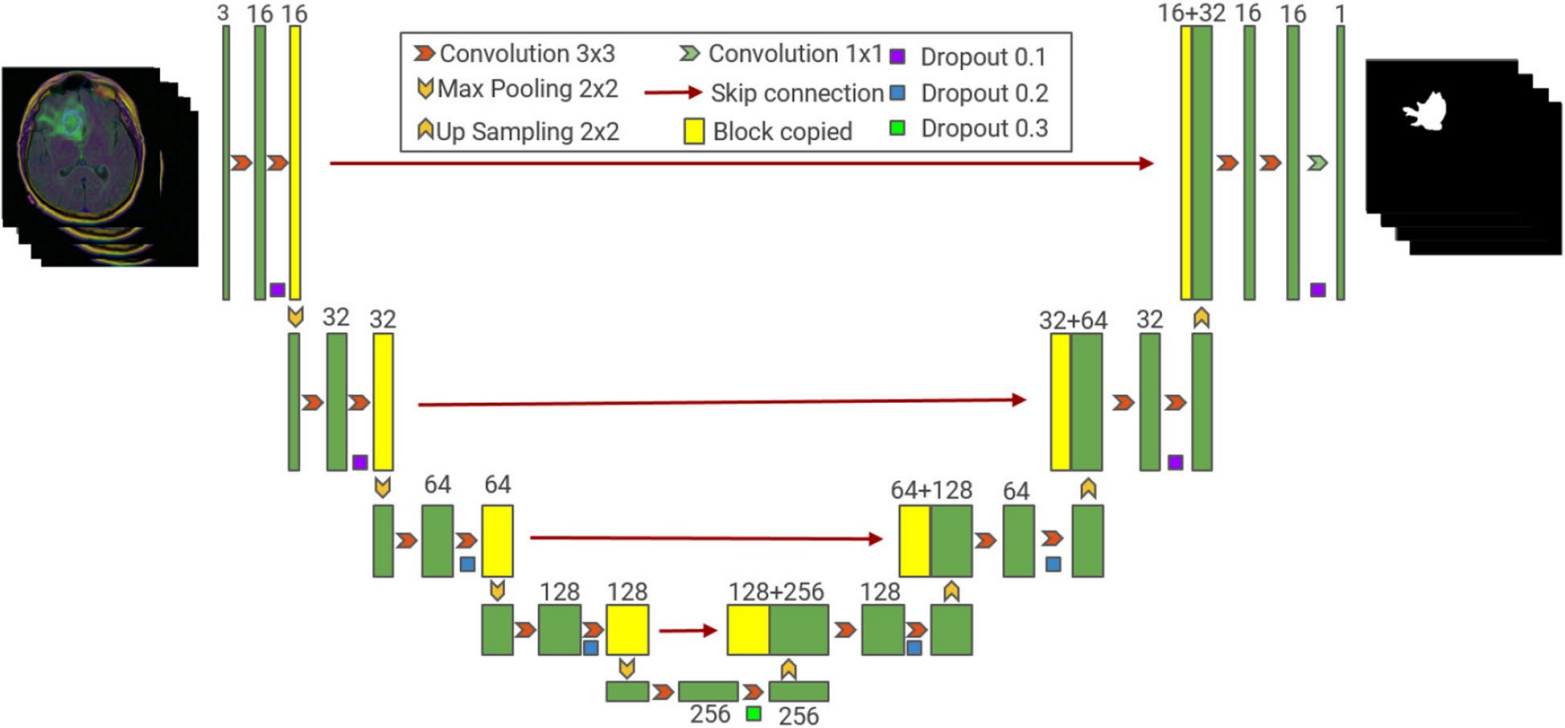
Network structure of U-Net model

U-Net was proposed by Ronneberger et al. in 2015. It is an improved model based on FCN. Initially, it was mainly used in the field of biomedical cell segmentation. It mainly adopts U-shaped symmetric structure of encoder (contraction path) and decoder (expansion path). As shown in Fig. 1, four down samples and four up samples are required. Each upsampling is fused with the feature output of the same size, and the feature information lost in the pooling process of the encoder is transmitted to the corresponding decoder at different stages using jump connections to enhance the feature information extraction capability. The network model is suitable for multiscale image segmentation; at the same time, it has the advantages of obtaining accurate segmentation results and fast segmentation speed in the case of a small amount of data training model.

Because the location and size of tumors in different images vary greatly, the network needs to have enough receptive fields and powerful spatial multi-scale processing capabilities.

Due to the large differences in tumor location and size in different images, the network needs to have sufficient receptive field and powerful spatial multi-scale processing capability. However, unlike Atrous Spatial Pyramid Pooling (ASPP), U-Net cannot master as much semantic information of different scales as possible, nor can it satisfy the accurate segmentation of the region near the target edge in the image [17]. Therefore, the FPN structure is mainly added to U-Net in this paper to improve U-Net's ability to integrate multi-scale semantic information and enrich the information contained in the features used in pixel tag classification.

### FPN model

**Fig. 2:**
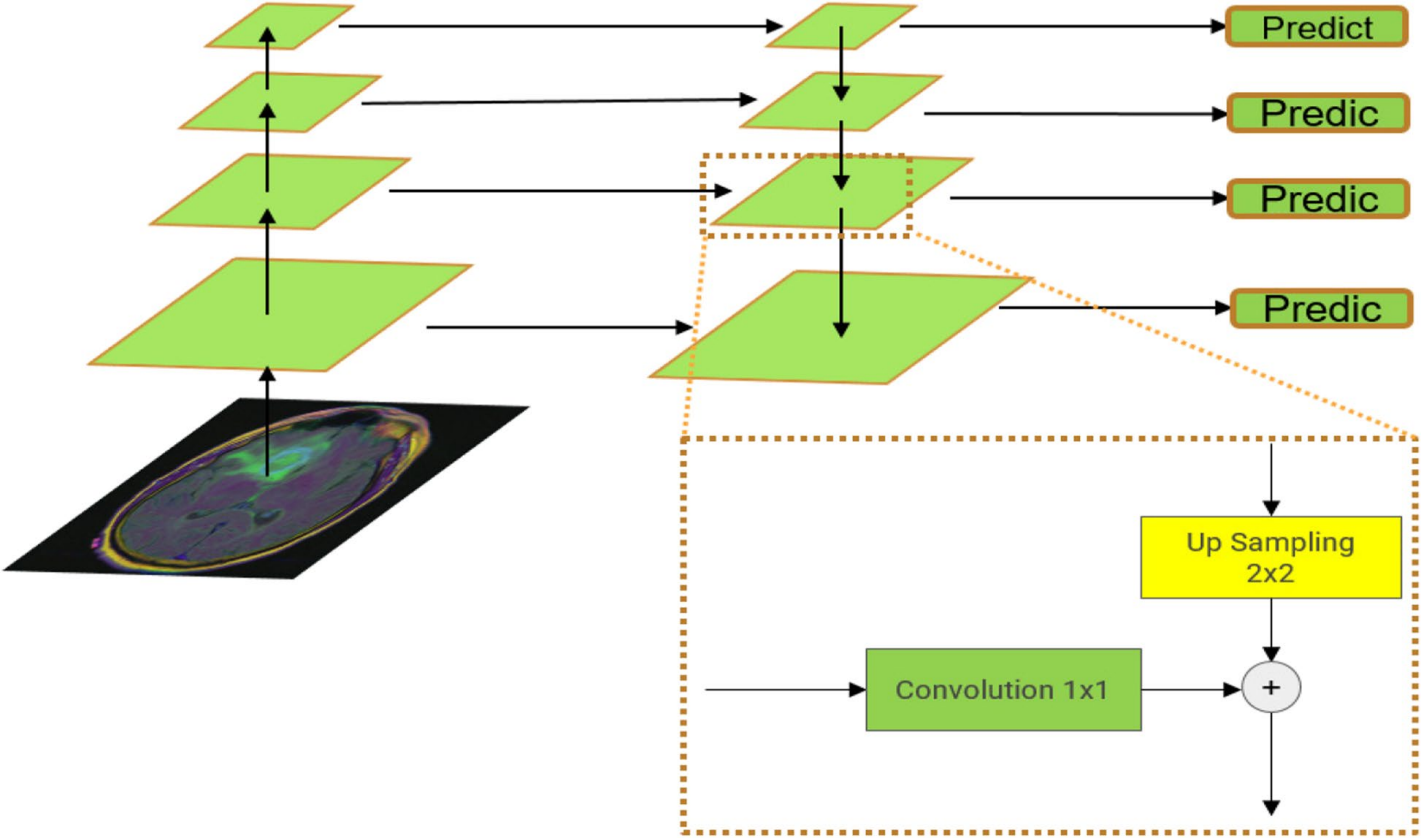
Network structure of FPN model

FPN structure is called feature pyramid network, which is widely used in target detection. FPN structure can effectively integrate multi-scale semantic information from encoders. FPN consists of three main parts: bottom-up process, top-down process and horizontal connection. As shown in Fig. 2.

In the bottom-up pathway, as one moves up, the spatial resolution decreases while high-level structure features extracted increase. At the top of the bottom-up pathway, 1 × 1 convolution is used to reduce the channel depth. Then two 3 × 3 convolutetion were applied which gives the first feature map for segmentation. In the top-down pathways, as one goes from the top-down, the previous layer is up sampled by using nearest neighbors up sampling. Again, 1 × 1 convolution is applied to the corresponding feature maps in the bottom-up pathway and then added element-wise. Two 3 × 3 convolutions are then applied to output the final feature map for image segmentation. In the end, all feature maps having 128 channels are concatenated resulting in 512 channels. A 512 3 × 3 convolution filter is then applied with batch normalization and RELU activation and then a 1 × 1 convolution is applied to obtain a final feature map.

### Improved FPN model

**Fig. 3:**
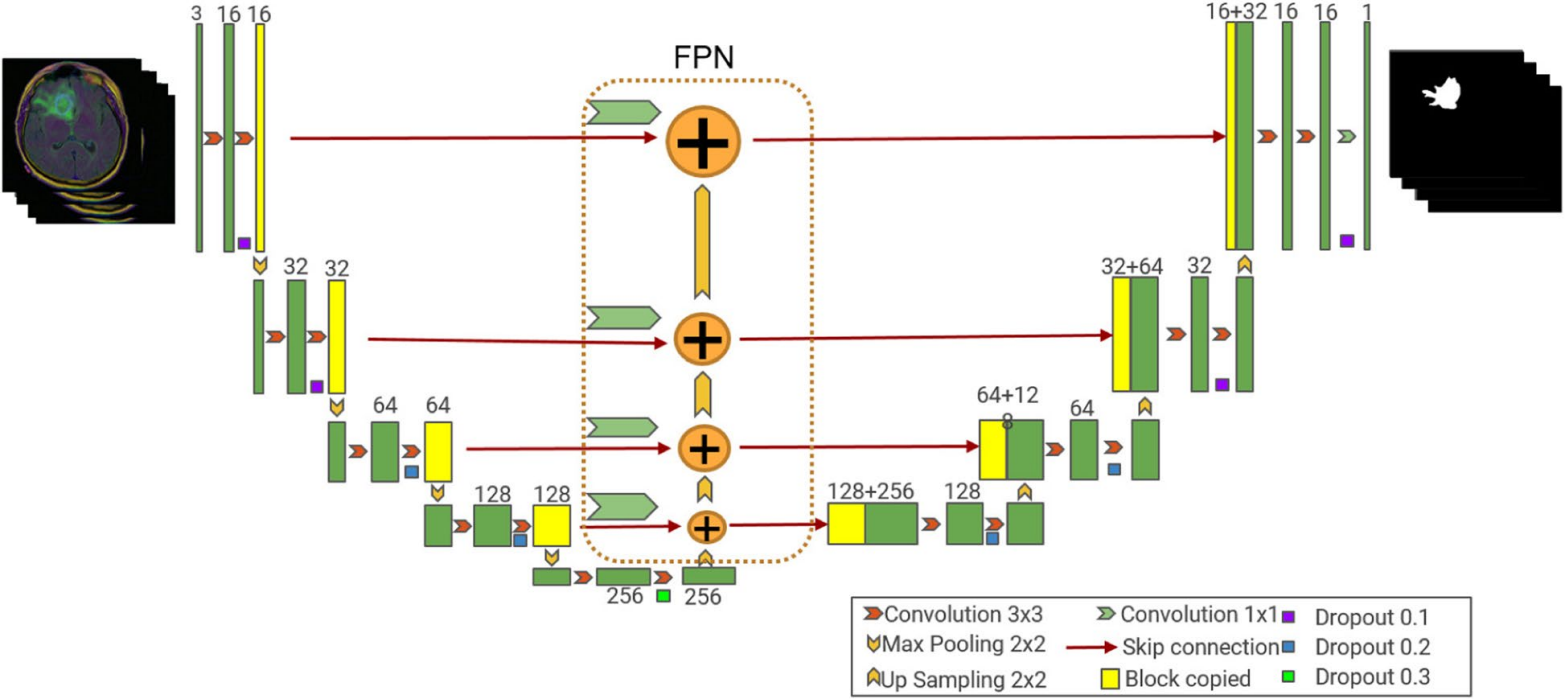
Network structure of improved FPN model

In order to improve the segmentation effect of brain tumors, this paper improved the U-Net model, introduced FPN structure into U-Net structure, and made full use of the multi-scale information ability of U-Net encoder [18]. The deconvolution oversampling used in U-Net can obtain relatively smooth structural features, but the original features are not well preserved after the transformation of convolution and nonlinear activation layer [19]. The FPN oversampling method uses quadratic linear interpolation, which preserves the original features more completely than deconvolution operation. The integration of additional functionality ensures that each level passed to the decoder contains as much multi-scale information as possible. By comparing the structure of FPN and U-Net, it is found that they are similar. The horizontal connection in FPN structure can be realized through the horizontal connection in U-Net. This capability facilitates the extension of the U-Net model using the FPN structure. While making full use of U-Net structure, the information of different scales contained in U-Net model is widely used. The network structure of the improved FPN model is shown in Fig. 3.

### Dataset

**Fig. 4:**
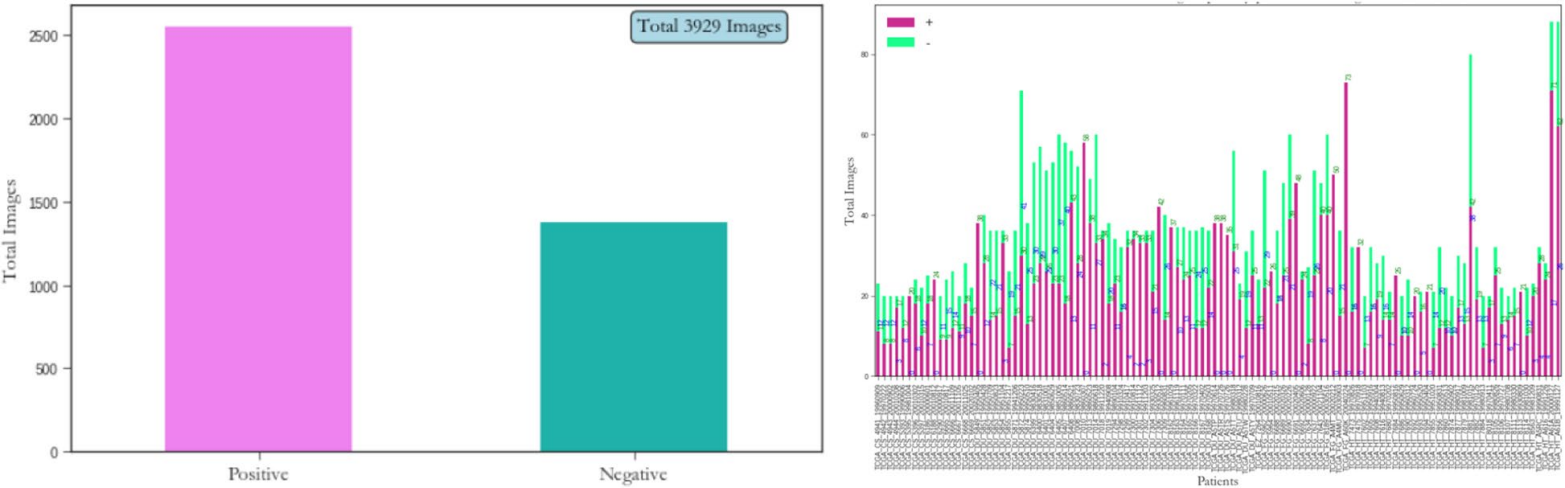
Histogram distribution of diagnostic datasets

**Fig. 5:**
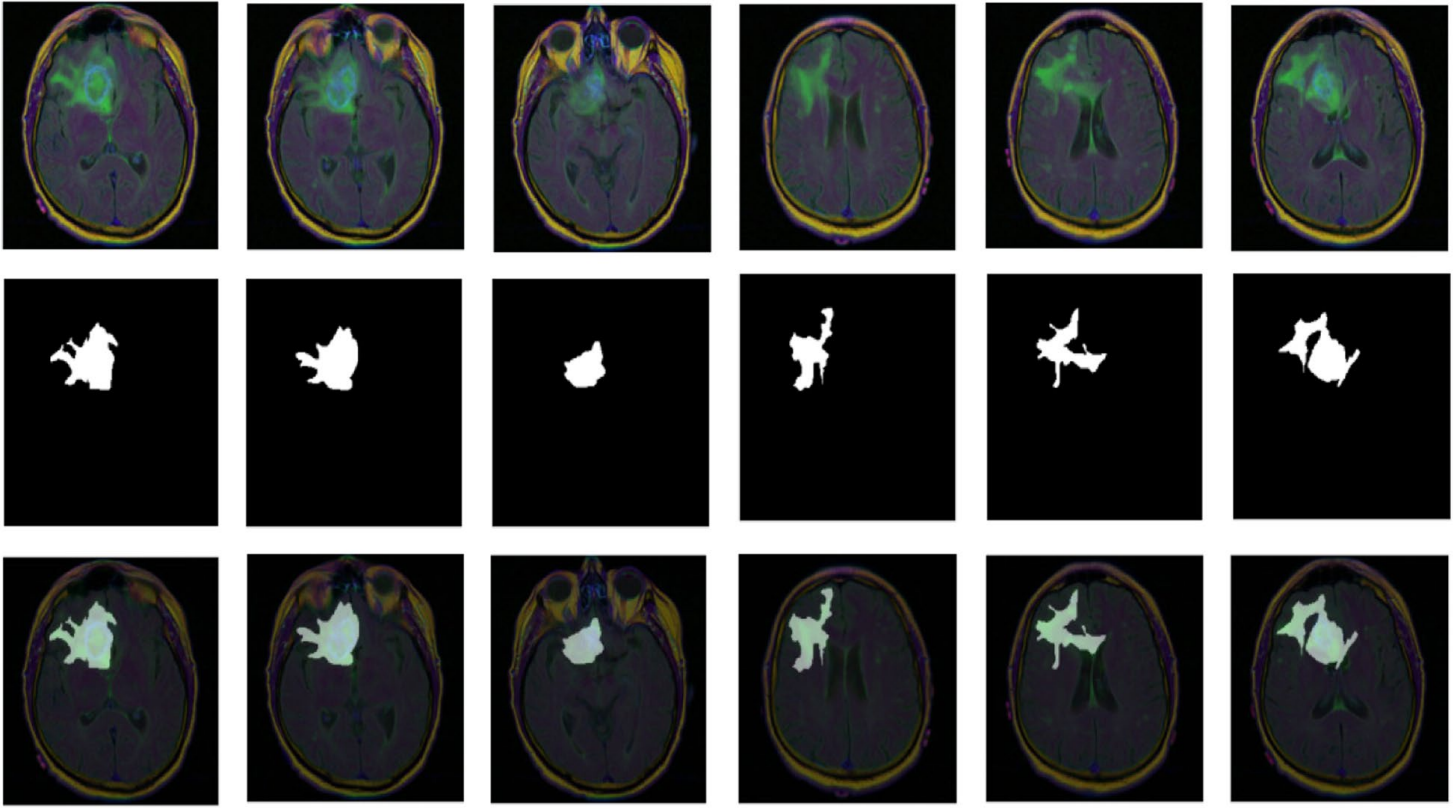
Dataset visualization (MRI image above, segmentation mask in the middle, and fusion image of original image and segmentation mask below)

**Fig. 6:**
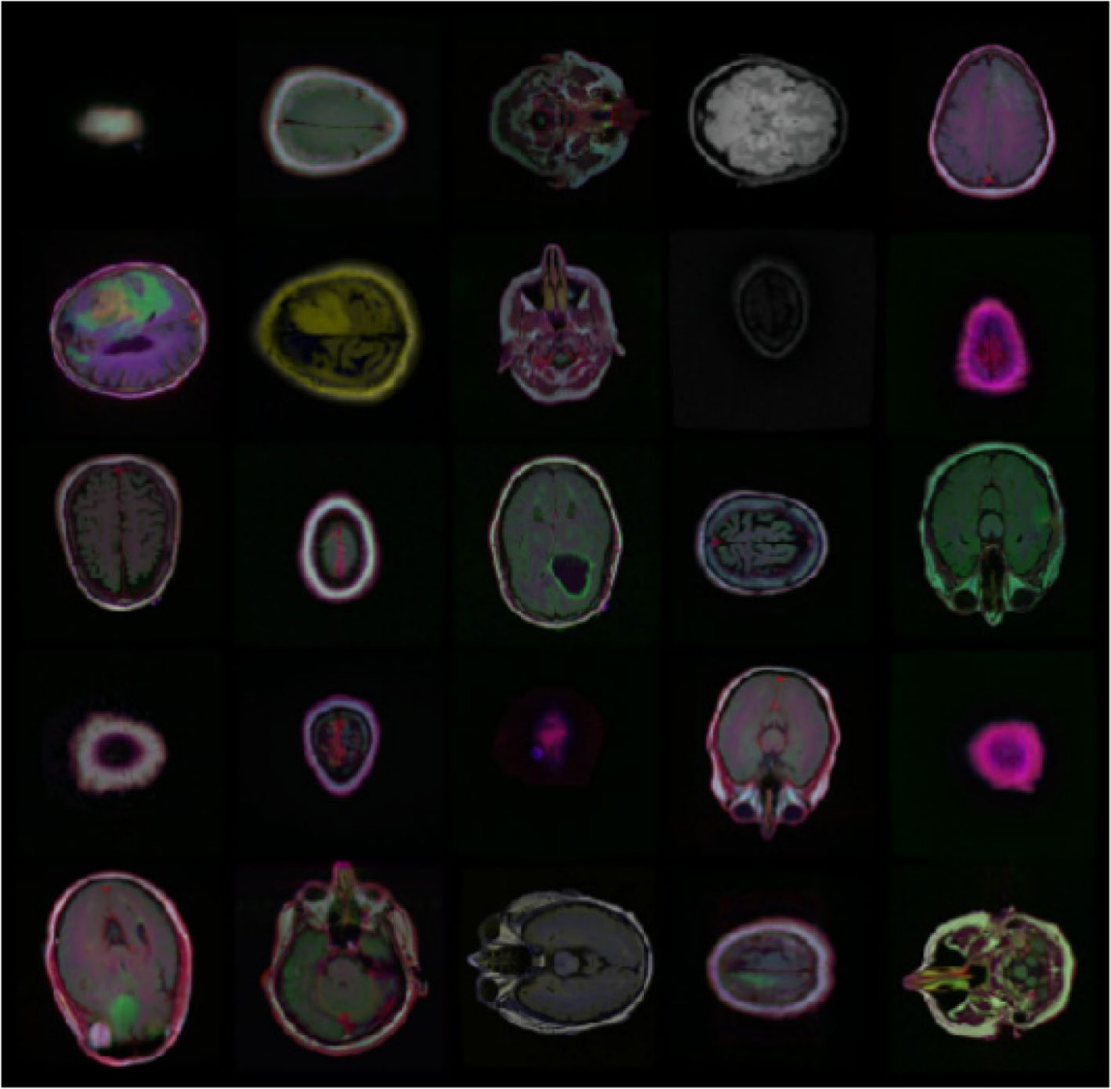
Brain tumor images of different enhancement types

The MRI data set used in this paper was derived from publicly available medical imaging data from the cancer genome atlas (TCGA) [20]. A total of 110 patients with an average age of 47 were enrolled in the TCGA database, including 56 males and 53 females, including 1 unidentified patient, all of whom were patients with lower-grade glioma (LGG). The data consisted of 3,929 MRI images, including 1,373 images with brain tumors and 2,556 normal brain images without tumors. The histogram distribution of MRI diagnostic data set is shown in Fig. 4. A positive sign indicates a tumor in the MRI image, while a negative diagnosis indicates the presence or absence of a tumor in the MRI image. Figure 5 shows the distribution of the diagnostic data sets (positive and negative) for each patient. Figure 6 Visualizes the brain tumor dataset with a tumor mask, shown from top to bottom as: original brain MRI, corresponding segmentation mask, and fusion images of the original image and segmentation mask. The MRI image is a 3-channel RGB image, the corresponding segmentation mask is a single-channel grayscale image, and the image resolution is 256X256.

### Data preprocessing

In the field of computer vision, data preprocessing plays a very important role, especially in medical image analysis, redundant information and inaccurate data may reduce the performance of the model. In this paper, data samples are preprocessed in four ways: data filtering, global pixel normalization, data segmentation and data enhancement. First, in order to alleviate the problem of imbalance between positive and negative sample categories in training samples, images without brain tumors are deleted, and the resulting dataset has 1373 images and 1373 corresponding tumor masks. Then, the global pixel normalization processing is performed on the image, and the pixel value is scaled from 0 to 255 to 0 to 1, so that the convergence speed is faster when the gradient drops, so as to better train. After the segmentation mask is normalized, it becomes a binary mask through threshold processing, that is, the background pixel (tumor free area) is 0, and the foreground pixel (tumor area) is 1. Training set, verification set and test set are divided into 70%, 20% and 10%. The segmented training set has 988 images, the verification set has 247 images, and the test set has 138 images. Deep learning requires a large number of labeled datasets, but medical datasets are limited. To avoid over fitting and improve the generalization ability of the model, we use data enhancement technology [21, 22]. Data enhancement is a method of scaling, flipping, and rotating existing data while retaining the same label. In this study, we used six enhancement methods: horizontal offset, transpose processing, vertical offset, blur processing, random clipping, random rotation, random scaling, random flipping, and random brightness. Table 1 lists the types and parameters of data enhancement. Figure 6 Brain tumor images with different enhancement types.

**Table 1:** Types and parameters of data enhancement

Serial number	Type of augmentation	Parameters
1	Horizontal shift	0.25
2	Transpose	0.25
3	Vertical shift	0.25
4	Blur	0.05
5	Random Crop	0.05
6	Random Rotate	0.5
7	Random Resize	0.25
8	Random flip	0.5
9	Random brightness	1.0

### Performance assessment metrics

In the medical image segmentation task, due to the small region of interest (ROI), the pixels of the positive sample occupy a relatively small proportion in the whole image. Using cross entropy loss function for training will make the model focus on the learning of negative samples, thus affecting the segmentation effect of positive samples. Therefore, Dice loss is adopted in this paper to solve the problem of disequilibrium between classes. Dice is a set similarity measurement index, which is usually used as a measurement index in medical image competition. The value range of Dice coefficient is [0,1], and the value of Dice 1 is an extreme condition, indicating that the predicted value of network is exactly the same as the label graph. When Dice is 0, the predicted network value is completely different from the label graph. The formula is as follows: where, A represents the set of pixels actually segmented; B represents the set of truly segmented pixels in the tag; ξ is a parameter set to prevent denominator being 0, ξ in this experiment is 100.

$${L}_{dice}=1-\frac{2\times \left|A\bigcap B\right|+\xi }{\left|A\right|+\left|B\right|+\xi }$$

### Evaluation indicator

This paper uses three evaluation indexes to evaluate the model's performance, which are dice similarity coefficient (DSC), Jaccard index, also known as IoU index and accuracy (ACC). Each evaluation index is defined as follows: Where, A represents the set of pixels actually segmented; B represents the set of truly segmented pixels in the tag; TP, TN, FP and FN correspond to true positive (labeled 1 with a predictive value of 1), true negative (labeled 0 with a predictive value of 0), false positive (labeled 0 with a predictive value of 1) and false negative (labeled 1 with a predictive value of 0) respectively.

$$DSC=\frac{2\times \left|A\bigcap B\right|}{\left|A\right|+\left|B\right|}$$

$$Jac/IoU=\frac{\left|A\bigcap B\right|}{\left|A\right|+\left|B\right|-\left|A\bigcap B\right|}$$

$$Acc=\frac{\mathrm{TP}+\mathrm{TN}}{\mathrm{TP}+\mathrm{TN}+\mathrm{FN}+\mathrm{FP}}$$

### Training protocol

The experimental hardware environment was NVIDIA GeForce RTX Quadra K620 single GPU, and the software environment was Keras2.2.4 library at the back end of Tensorflow2.1. The input image size of the network model would be 256X256X3, the bitchsize would be set to 8, the number of training epoch would be set to 150, and the Early Stopping method would be used to stop the training in advance to avoid network overfitting. Hyperparameters such as filter size, learning rate, optimizer selection to optimize the segmentation results arebased on exhaustive methods of selection and then experiments are designed. The initial learning rate varied from 10^–3^ to 10^–4^ and based on the learning curve, the learning rate was selected to be 10^–4^.The Adam optimizer was used to update the parameters. In order to ensure the fairness of experimental results, all experiments were conducted under the same Settings above.

## Results

**Fig. 7:**
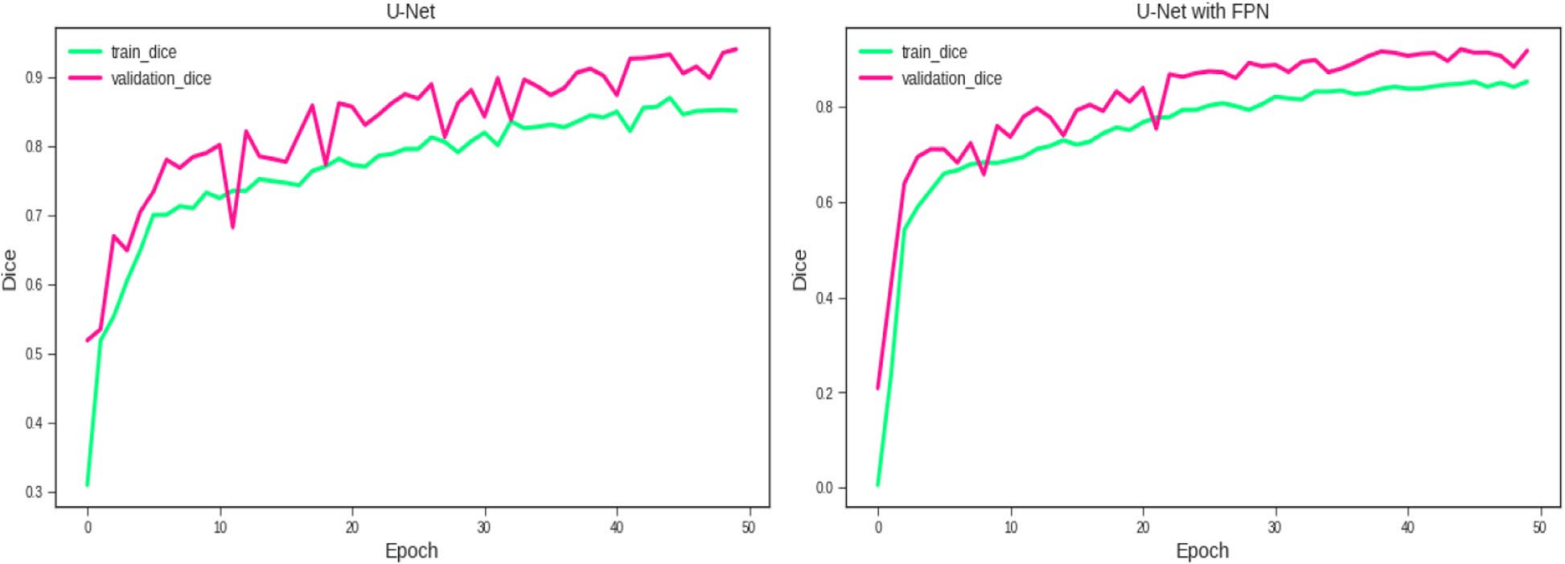
Change trend of brain tumor segmentation task during training

The algorithm proposed in this paper is based on the improvement of U-Net network and the improved strategy of FPN and data enhancement technology, which can effectively segment the brain tumor region. In order to intuitively and fairly evaluate the segmentation performance of the proposed algorithm, a comparison experiment was conducted with the classical algorithms U-Net and FPN. The performance comparison of the two segmentation algorithms is listed in Table 2. Figure 7 shows the variation trend and segmentation results of Dice in the training process. As can be seen from Table 2, the Dice coefficient, Jaccard index and accuracy of the improved FPN model proposed in this paper reached 92%, 86% and 99.1%, respectively, which were superior to the U-Net model. Experiments proved that the segmentation model proposed in this paper improved the segmentation accuracy of brain tumors. As can be seen from Fig. 7, the convergence rate of the improved FPN model is faster than that of the U-Net model, and it can be seen from the training trend of intact tumors that U-Net has a wide range of changes, indicating that the accuracy rate of segmentation results varies greatly during the training process.

**Table 2:** Performance evaluation of the two models

Model	DSC	JAC/IoU index	Accuracy
U-Net	89.6%	84%	98.7%
U-Net with FPN	92%	86%	99.1%

**Fig. 8:**
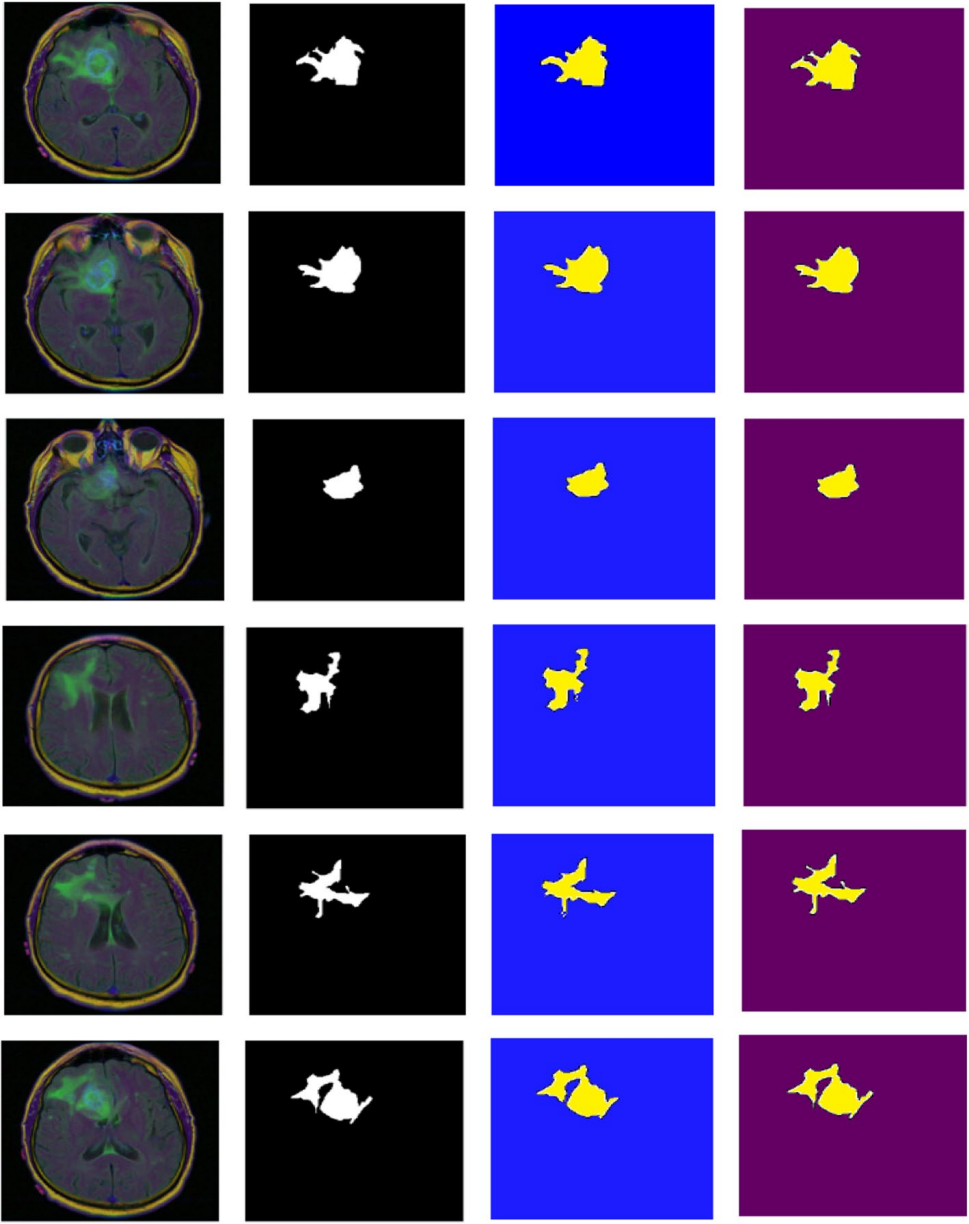
Segmentation results of two models (MRI image, mask, U-Net and improved FPN from left to right)

Figure 8 shows the visualization of brain tumor segmentation results by two segmentation methods. From left to right are the original brain MRI image, the corresponding segmentation mask, the segmentation results of U-Net model and the segmentation results of the improved FPN model proposed in this paper. As can be seen from Fig. 8, although the brain tumor region segmented by U-Net is relatively accurate, the segmented brain tumor boundary details are missing and the segmentation accuracy is not high, resulting in the problems of under-segmentation and over-segmentation. The algorithm proposed in this paper can segment most of the edge regions of the tumor well, which is closer to the segmentation template, and effectively refines the boundary of the brain tumor and enhances the detailed features of the outline of the brain tumor. It also improves the segmentation accuracy to some extent, indicating that the algorithm in this paper has strong segmentation ability.

Table 3 shows the comparison of the improved FPN model method with other paper methods and the performance of brain tumor segmentation on different data sets. The results show that the proposed method achieves the optimal performance with 92% Dice coefficient, 85% Jacc and 99.1% accuracy, which is close to or better than some existing algorithms in segmentation performance.

**Table 3:** Performance comparison of the proposed model with state-of-art methods

Literature	Dataset/Image Size	DSC	JAC/IoU index	Accuracy
(Dong N et al.,2017) [23]	10infants	85.03	-	-
(Pereira S et al.,2017) [24]	65subjects	88	-	-
(Zhao X et al.,2017) [25]	449patients	84	-	-
(Akkus Z et al.,2017) [26]	159images	-	-	87.7
(Hashemzehi R et al.,2017) [27]	TCGA	84		95
Our improve the FPN method	TCGA	92	86	99.1

## Discussion

This paper presents an improved FPN convolutional neural network for brain tumor image segmentation. On the one hand, the FPN module is used in the coding block to obtain enough receptive field and improve the adaptability of the model to different scale features. On the other hand, residual connections and data enhancement strategies are used to reduce the risk of overfitting and network degradation. The experiment used 1373 brain MRI images and the performance of all models was evaluated by Dice coefficient, Jacc index and accuracy. The U-Net model is compared with the method proposed in this paper. Experiments show that the proposed network structure can refine tumor boundaries and achieve high segmentation accuracy. The Dice coefficient was 92%, the Jaccard index was 86.%, and the accuracy was 99.1%. At the same time, different modules were added to U-Net for comparative ablation experiments. The results show that both the improved FPN model and the data enhancement technology can improve the model segmentation performance to a certain extent. Finally, the performance of the proposed method is compared with that of the most advanced method, and the competitiveness and superiority of the proposed method are illustrated. In the study of related issues, Sumit Tripathi [28] et al. attempted a total of 100 periods by optimizing the programs of Adam, SGD, and Adamax in the FPN algorithm, and then selected Adam based on the dice scores on the validation set. Since the cross entropy loss of dice continues to decrease on the verification set, the above training scheme helps to achieve the highest segmentation accuracy. The evaluation index values obtained for the network indicate that the algorithm works normally and provides good segmentation results. In conclusion, this study will provide new ideas for multi-scale research in the field of MRI segmentation of brain tumors.

## Data Availability

The data in this article adopt publicly available medical imaging data from the cancer genome atlas (TCGA).Download address: https://www.kaggle.com/datasets/mateuszbuda/lgg-mri-segmentation.

## Abbreviations

FCN: Full convolutional neural network
FPN: Feature pyramid networks
MRI: Magnetic resonance imaging
ASPP: Atrous spatial pyramid pooling
TCGA: The cancer genome atlas
LGG: Lower-grade glioma
ROI: Region of interest
ACC: Accuracy
DSC: Dice similarity coefficient
TP: True positive
TN: True negative
FP: False positive
FN: False negative
